# Supplementary material for: Forty new specimens of Ichthyornis provide unprecedented insight into the postcranial morphology of crownward stem group birds
Source: PeerJ. 2022 Dec 16;10:e13919. doi: 10.7717/peerj.13919 (PMC9762251; doi:10.7717/peerj.13919)
Supplement: Supplemental Information 2 — Includes Supplemental Table 1, Supplemental Figures 1-10 and Supplemental Trees 1-16 [file peerj-10-13919-s002.docx]

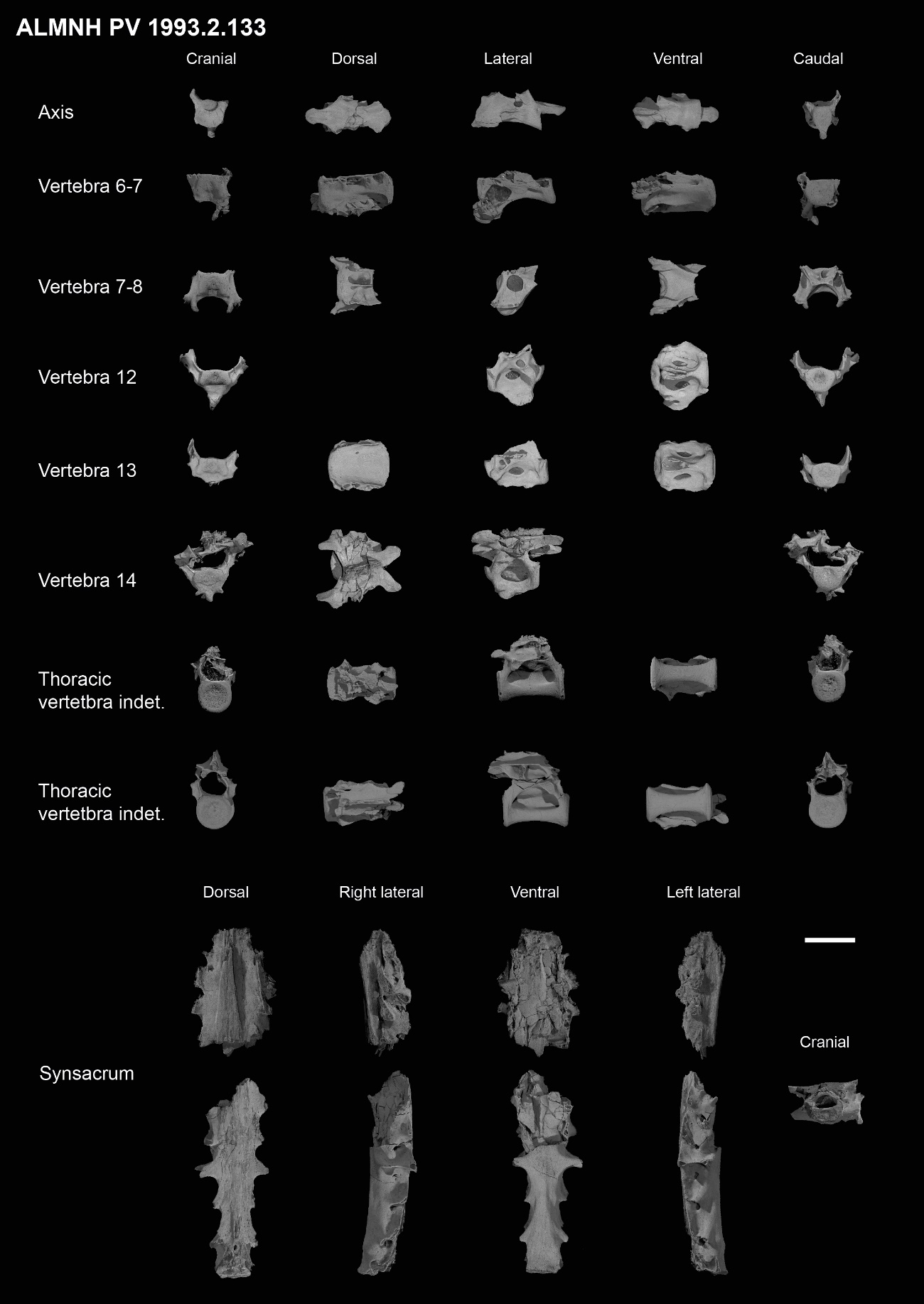


**Supplemental figure 1. Complete axial material from specimen ALMNH PV 1993.2.133.** Scale bar equals 1 cm.


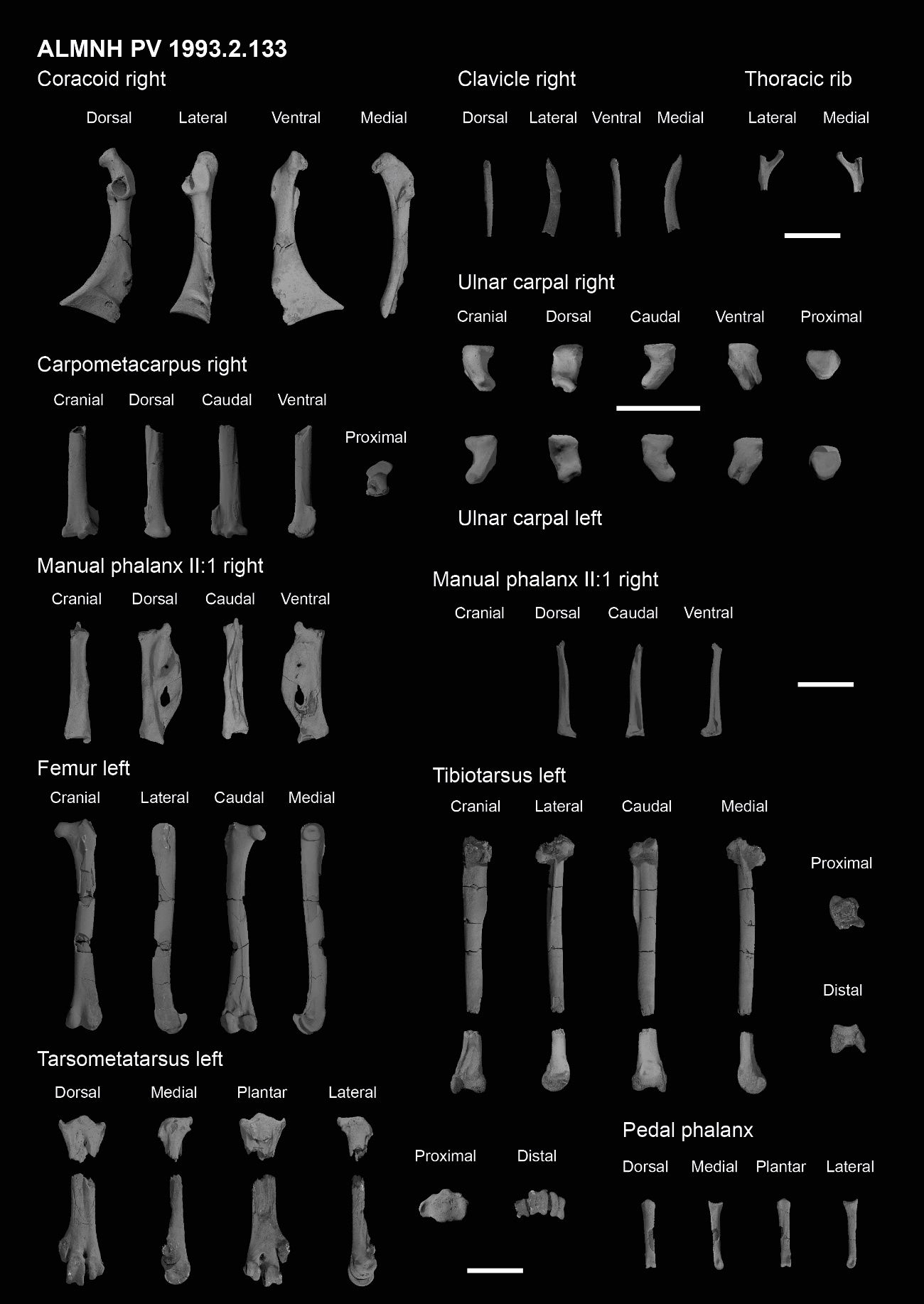


**Supplemental figure 2. Complete forelimb and hindlimb material from specimen ALMNH PV 1993.2.133.** Scale bars equals 1 cm.


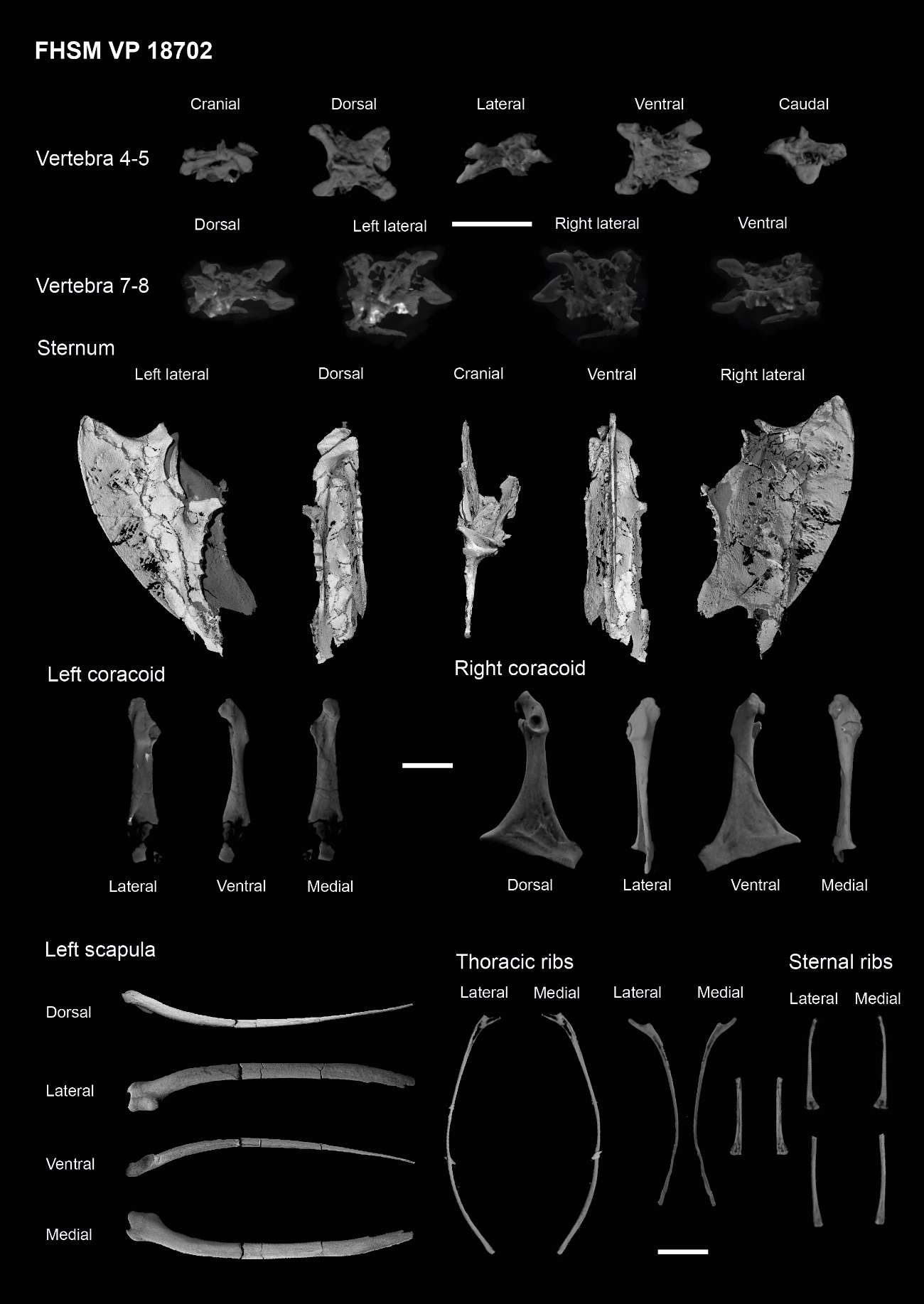


**Supplemental figure 3. Complete cervical, pectoral and thoracic material from specimen FHSM VP 18702.** Scale bar equals 1 cm.


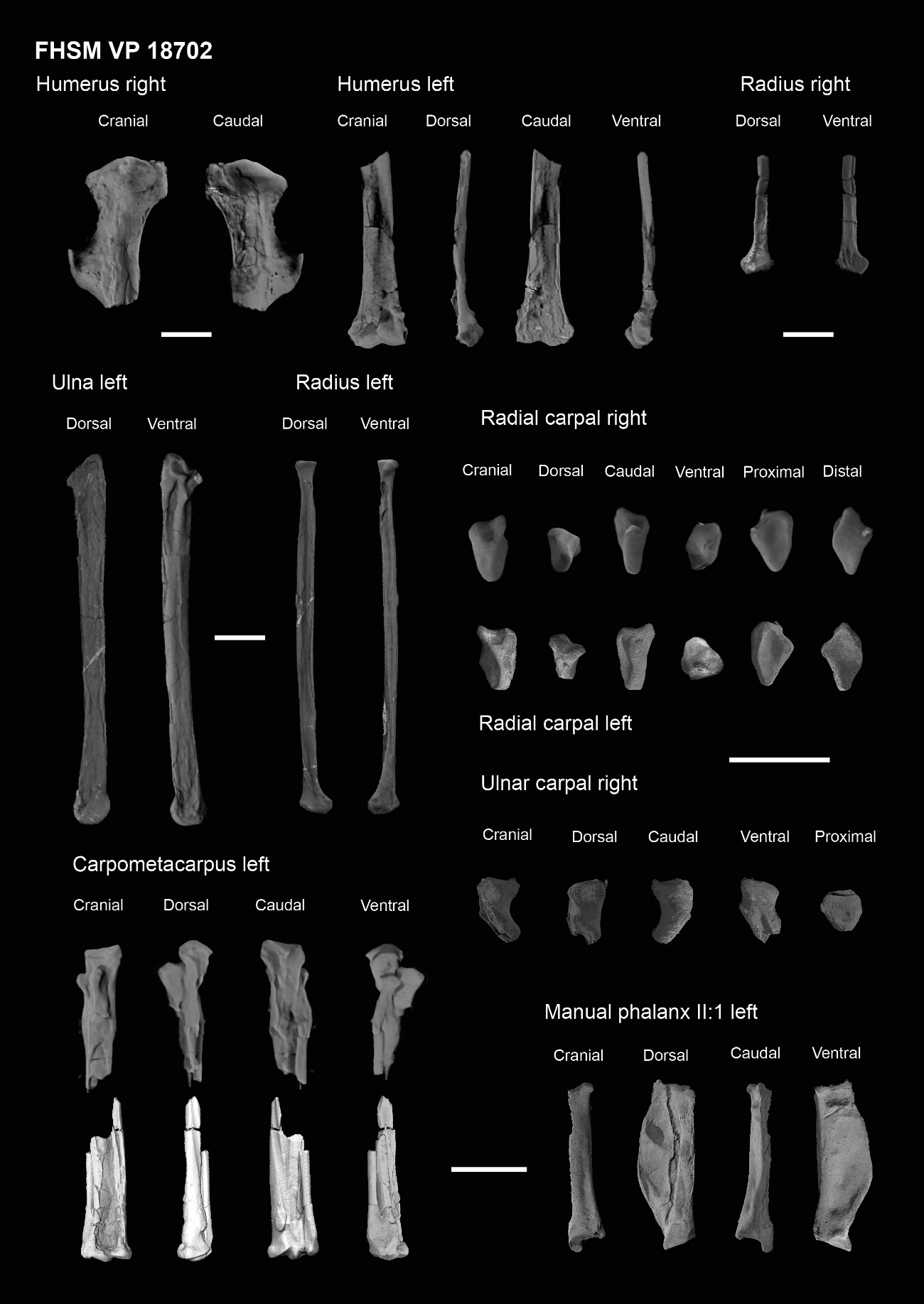


**Supplemental figure 4. Complete forelimb material from specimen FHSM VP 18702.** Scale bars equals 1 cm.


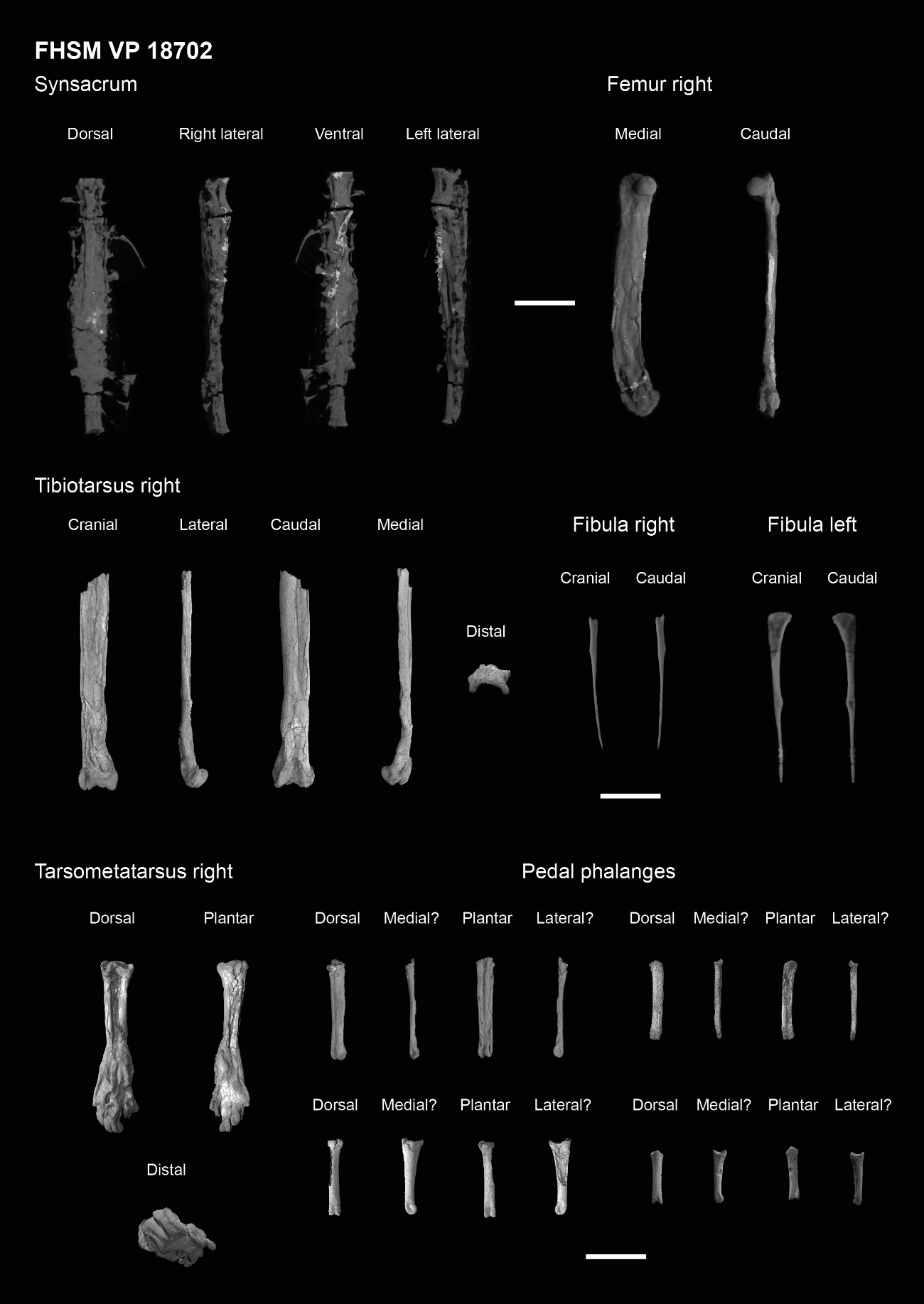


**Supplemental figure 5. Complete sacral and hindlimb material from specimen FHSM VP 18702.** Scale bars equals 1 cm.


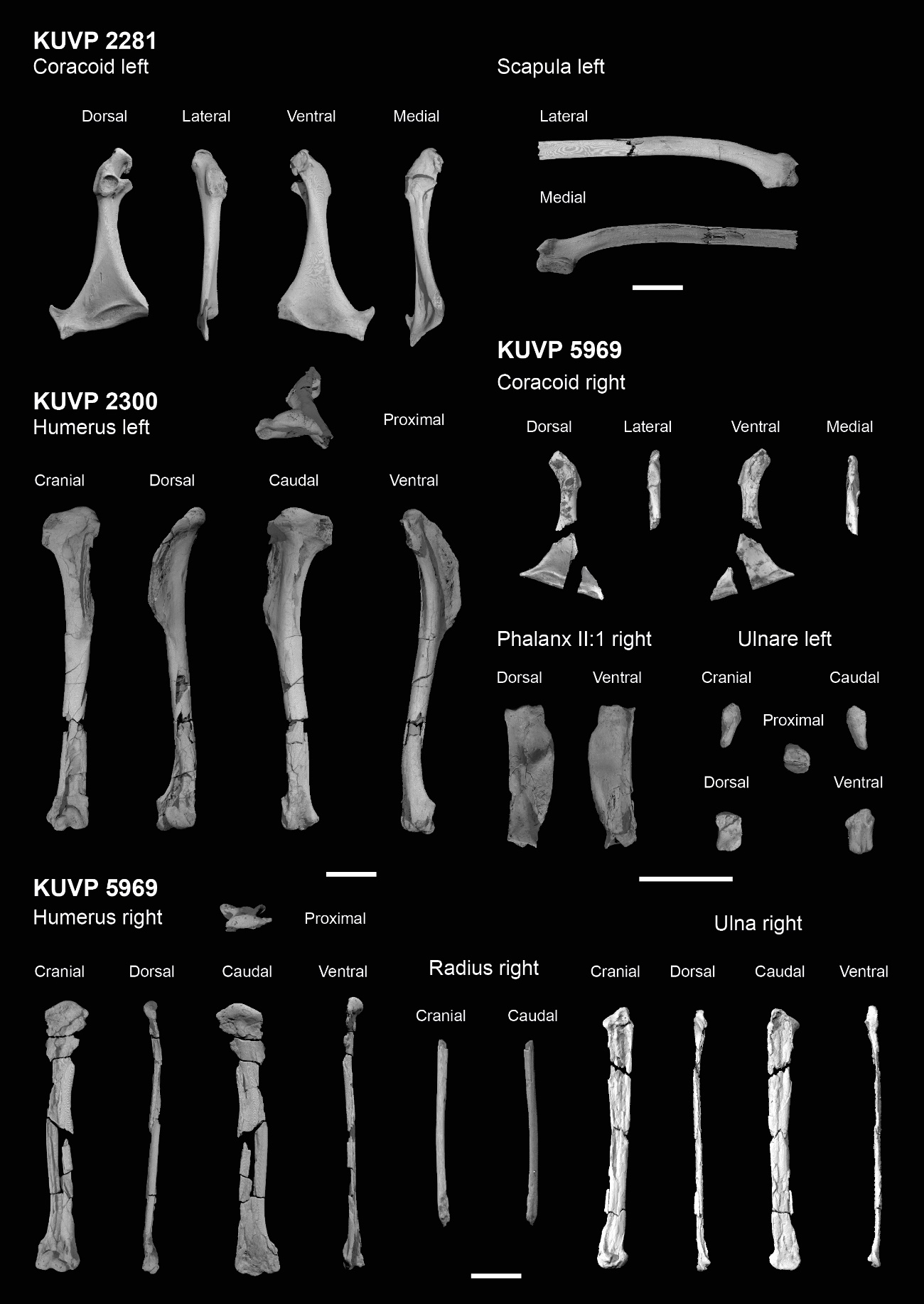


**Supplemental figure 6. Complete pectoral and forelimb material from specimens KUVP 2281, 2300 and 5969.** Scale bars equals 1 cm.

**
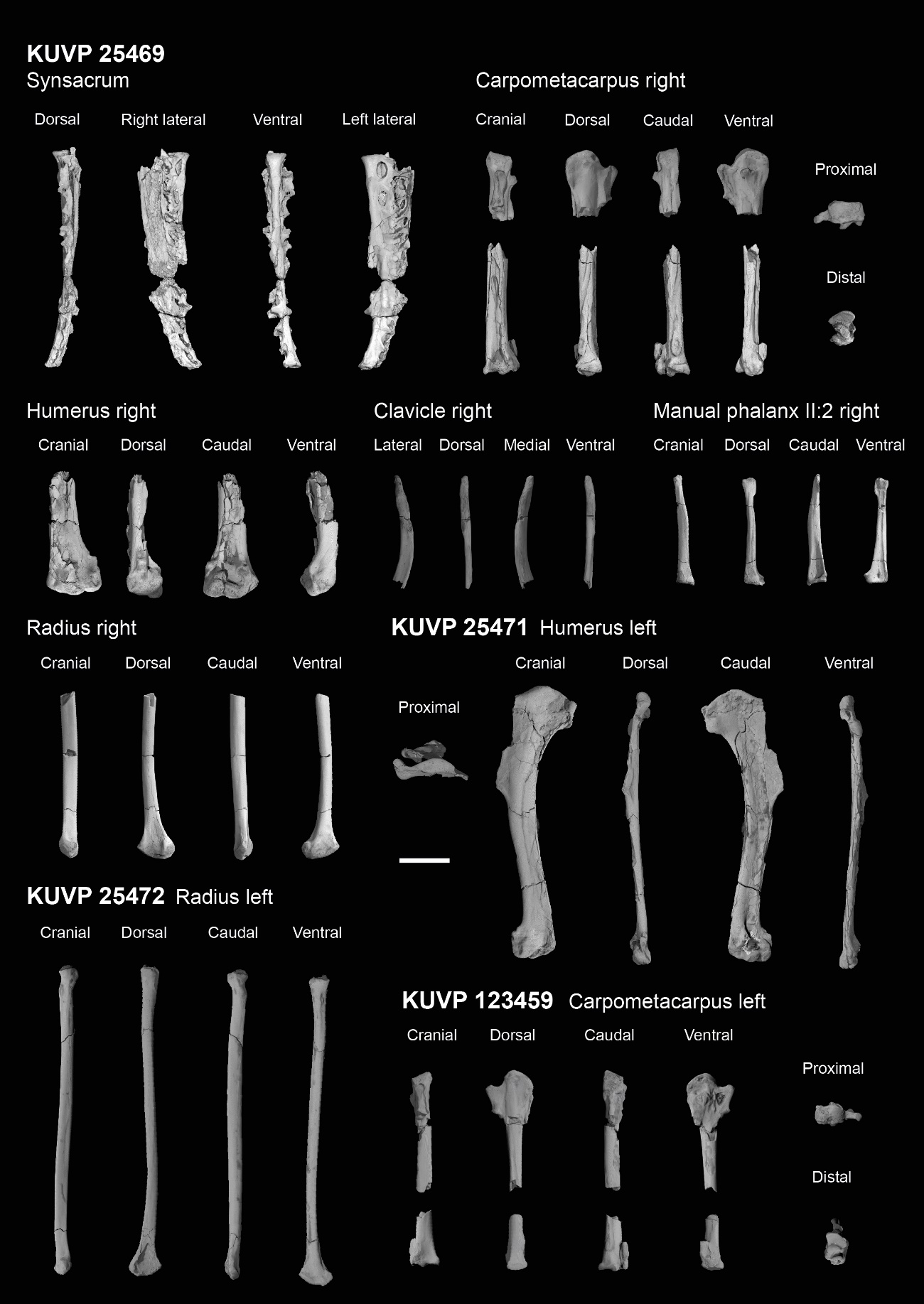
**

**Supplemental figure 7. Complete sacral, pectoral and forelimb material from specimens KUVP 25469, 25471, 25471 and 123459.** Scale bar equals 1 cm.


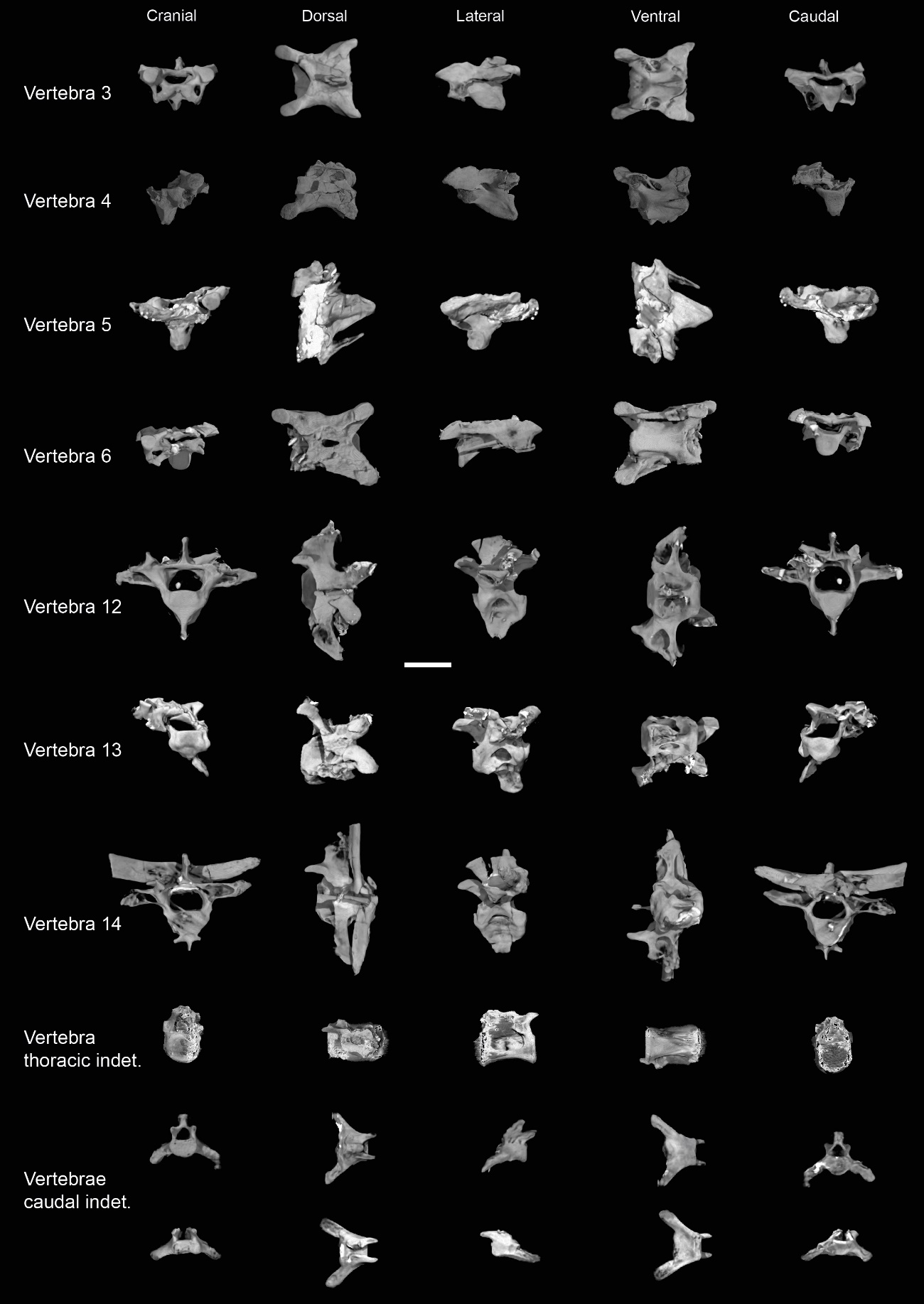


**Supplemental figure 8. Axial material from specimen KUVP 119673.** Scale bar equals 5 mm.


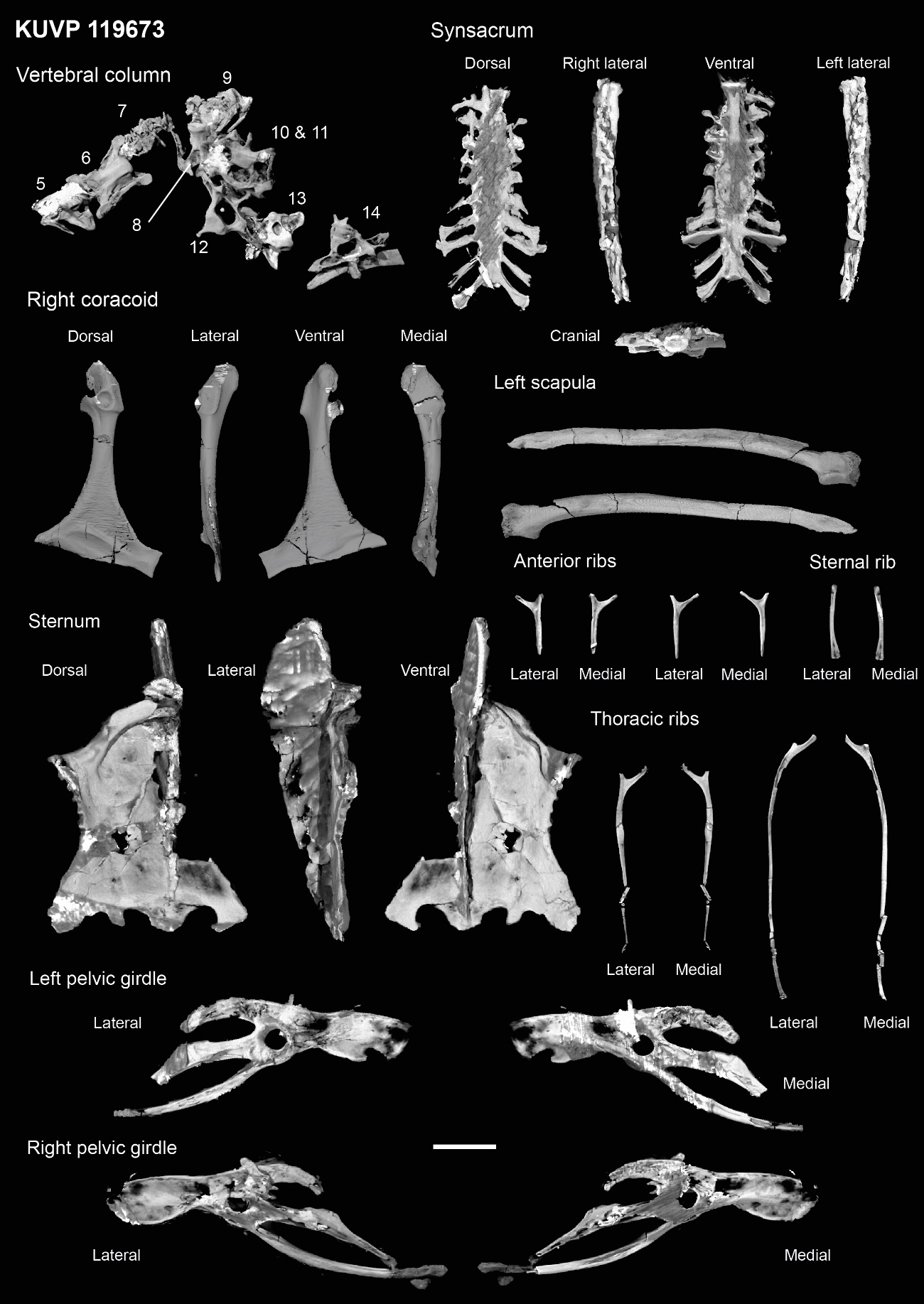


**Supplemental figure 9. Axial, pectoral, thoracic and pelvic material from specimen KUVP 119673.** Scale bar equals 1 cm.


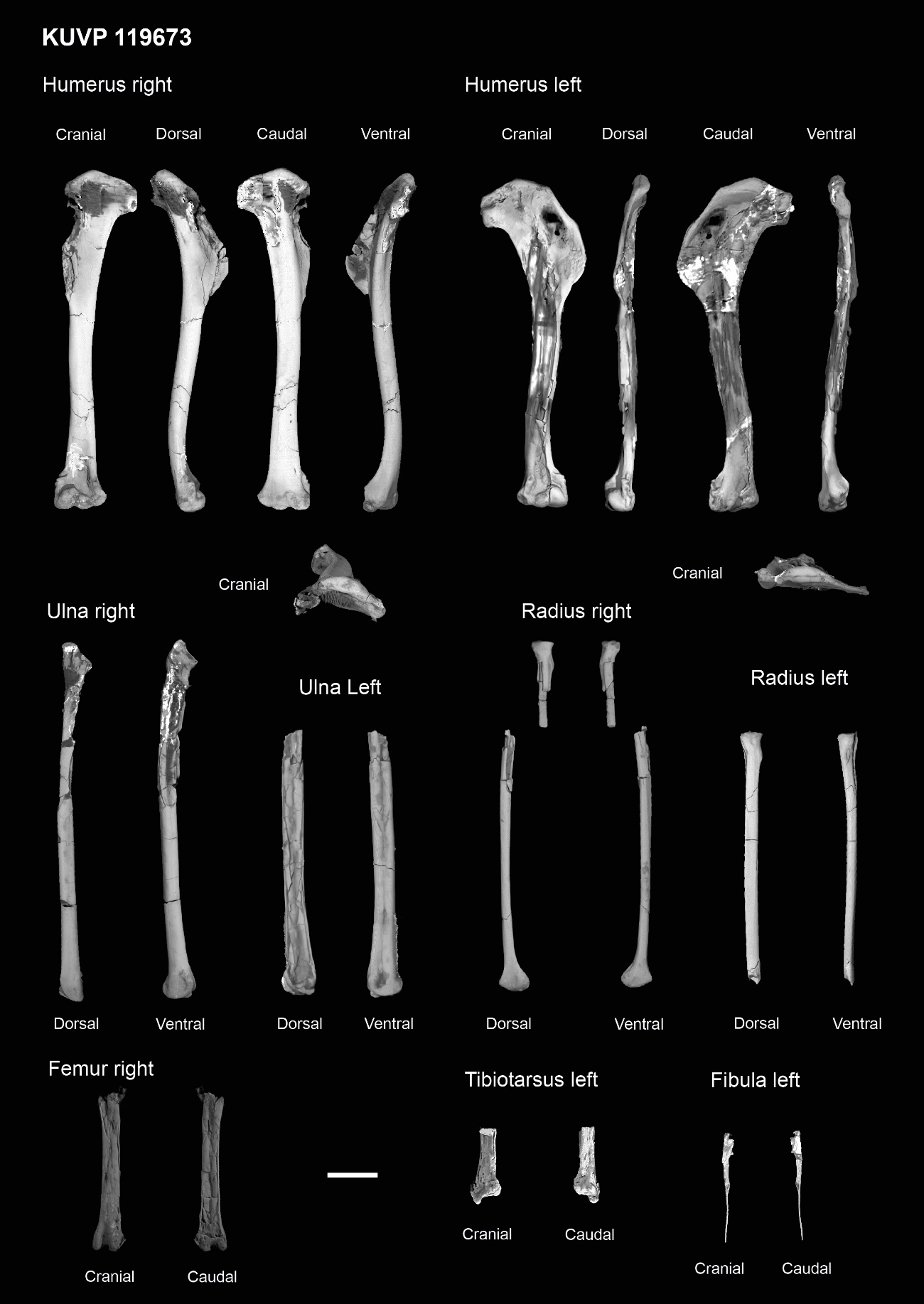


**Supplemental figure 10. Complete forelimb and hindlimb material from specimen KUVP 119673.** Scale bar equals 1 cm.


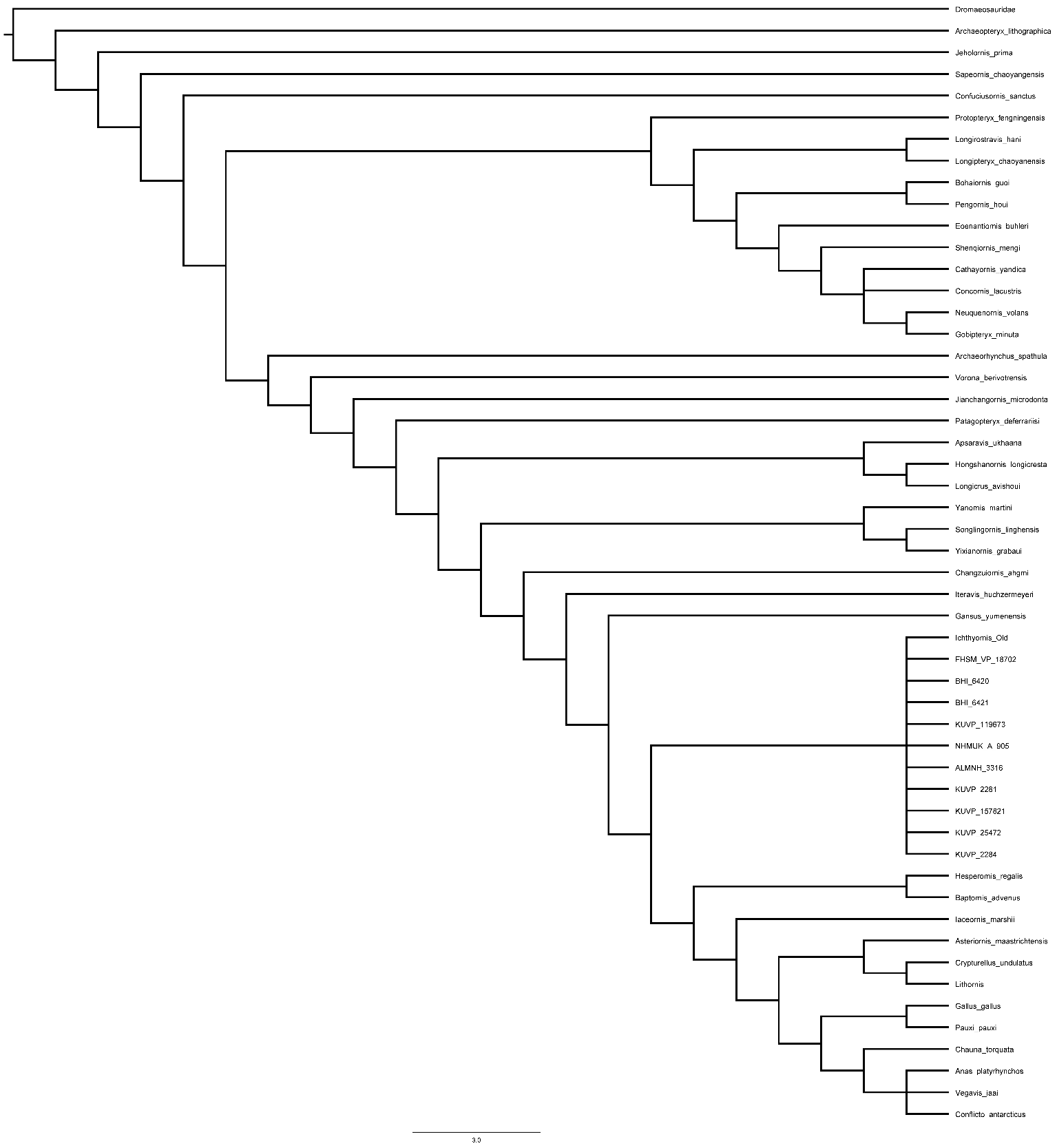
**Supplemental Tree 1**


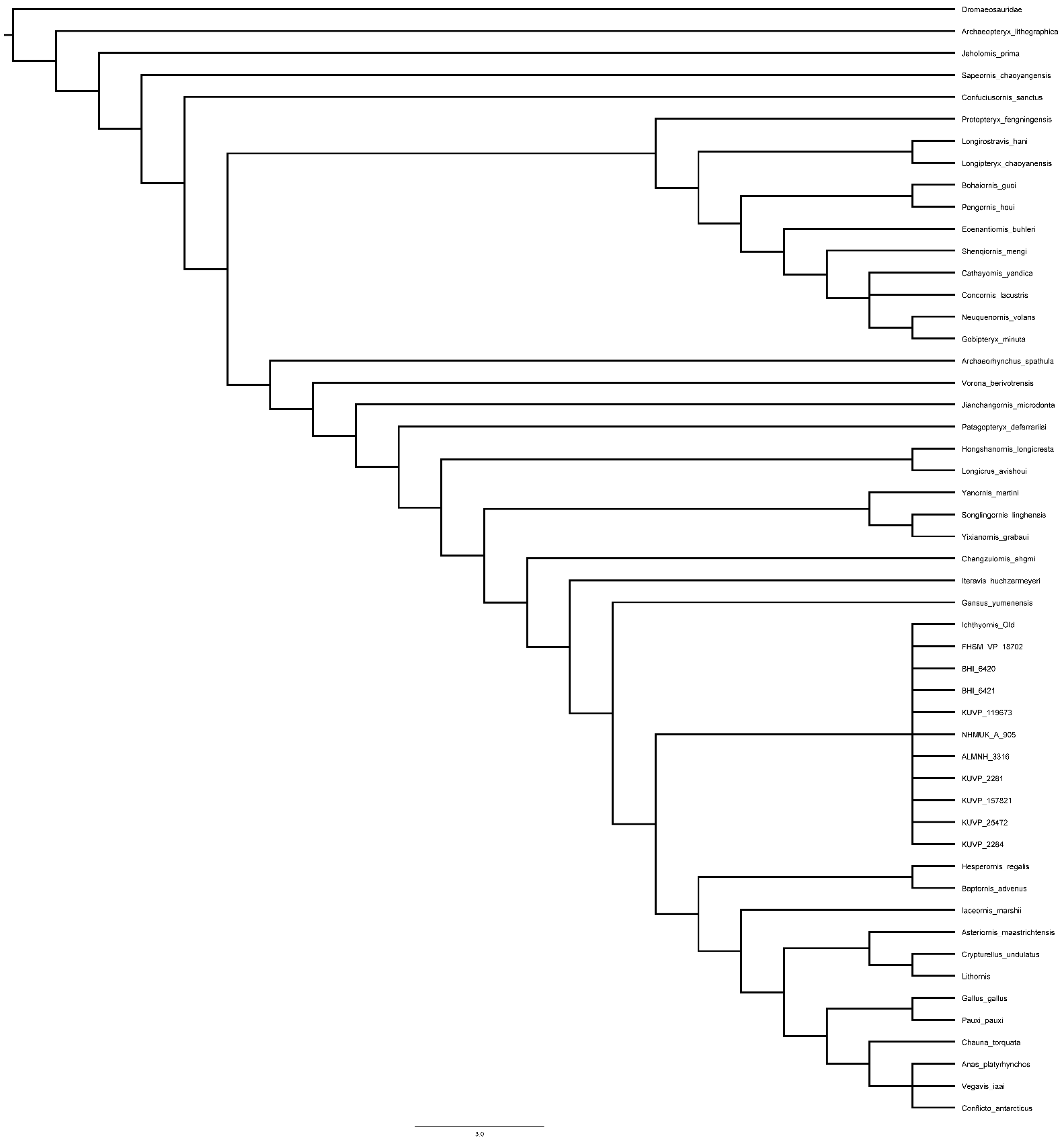
**Supplemental Tree 2**

**
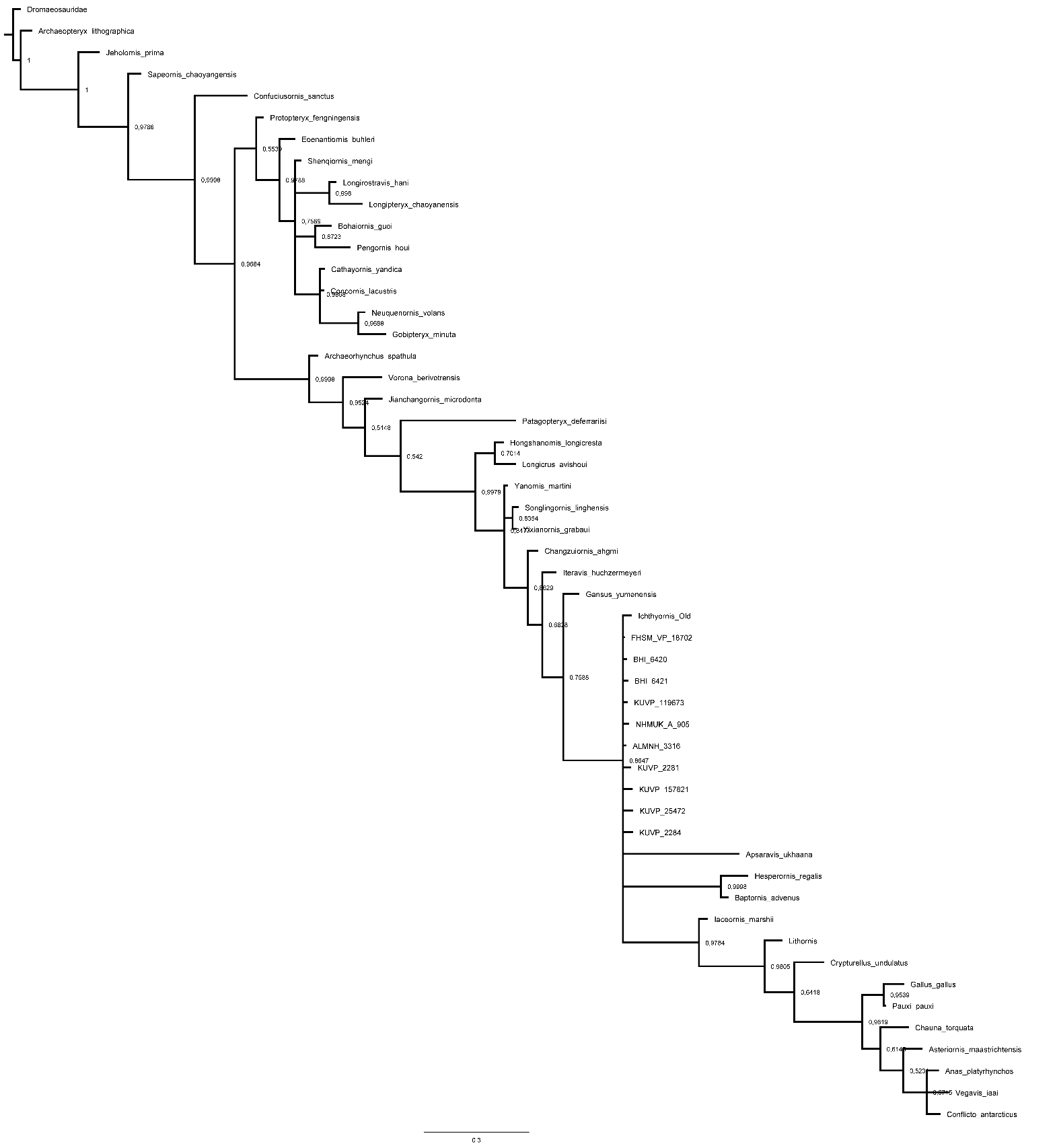
Supplemental Tree 3**

**Supplemental Tree 4**

**
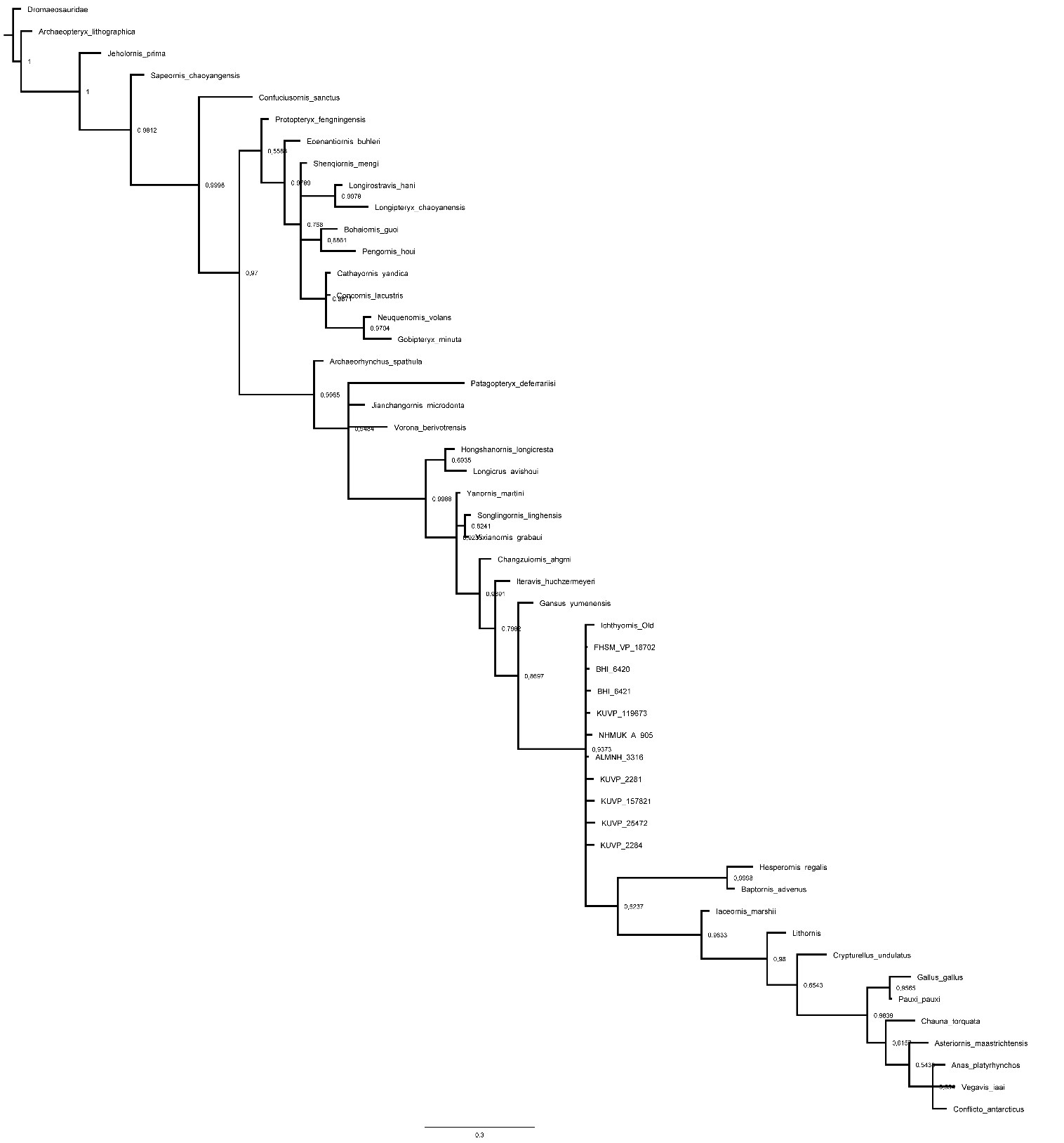
**


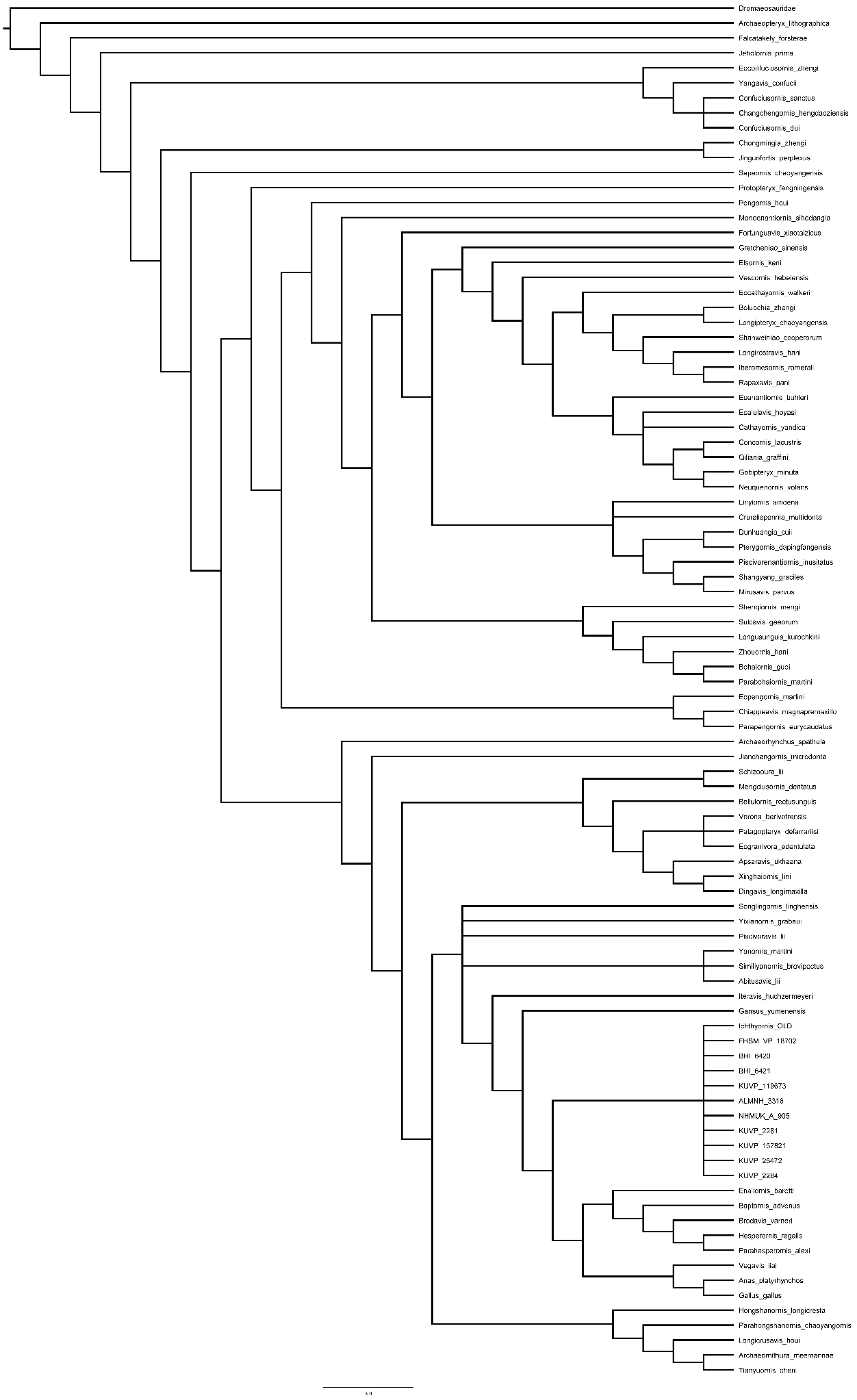
**Supplemental Tree 5**

**Supplemental Tree 6**

**
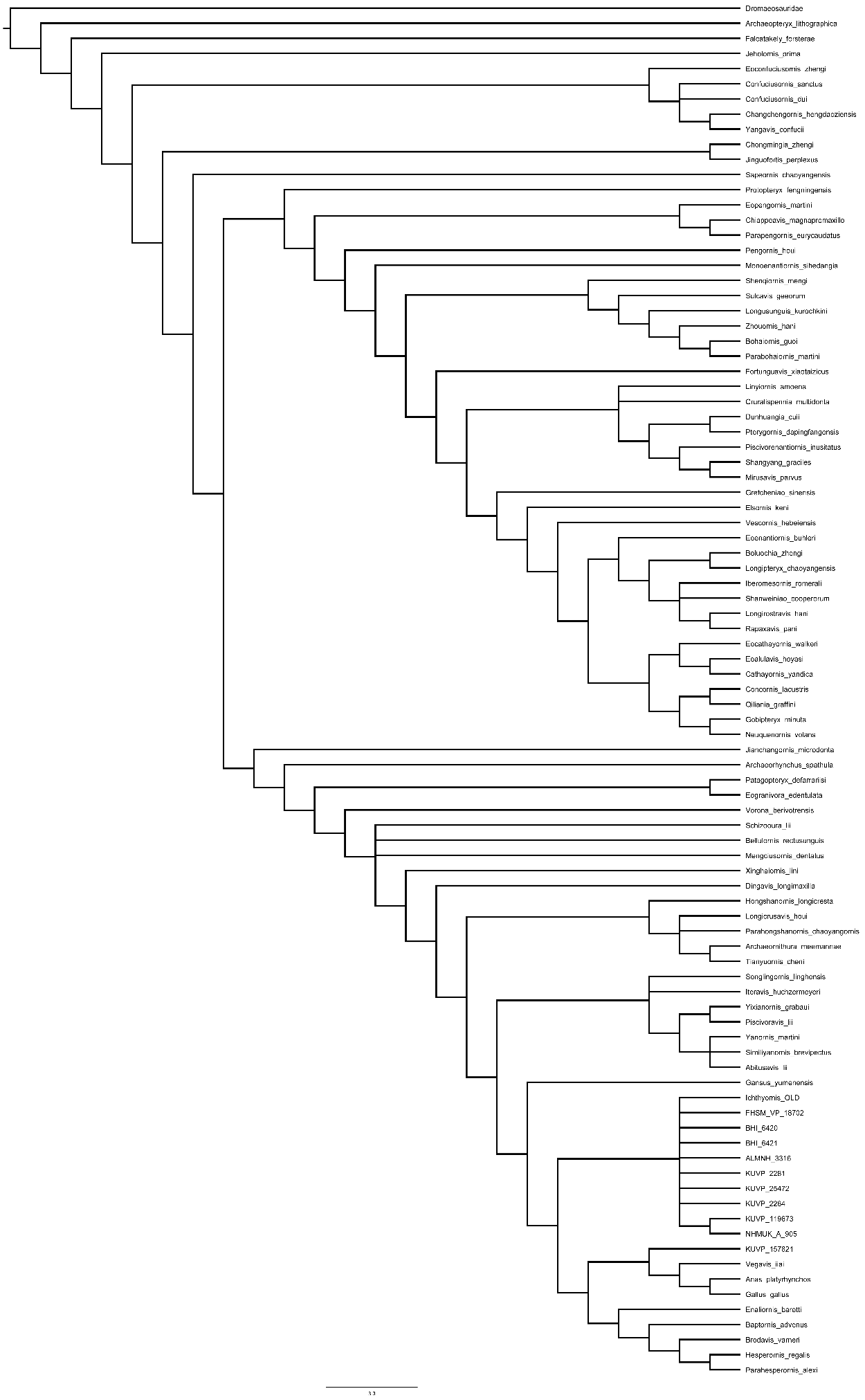
**

**Supplemental Tree 7**

**
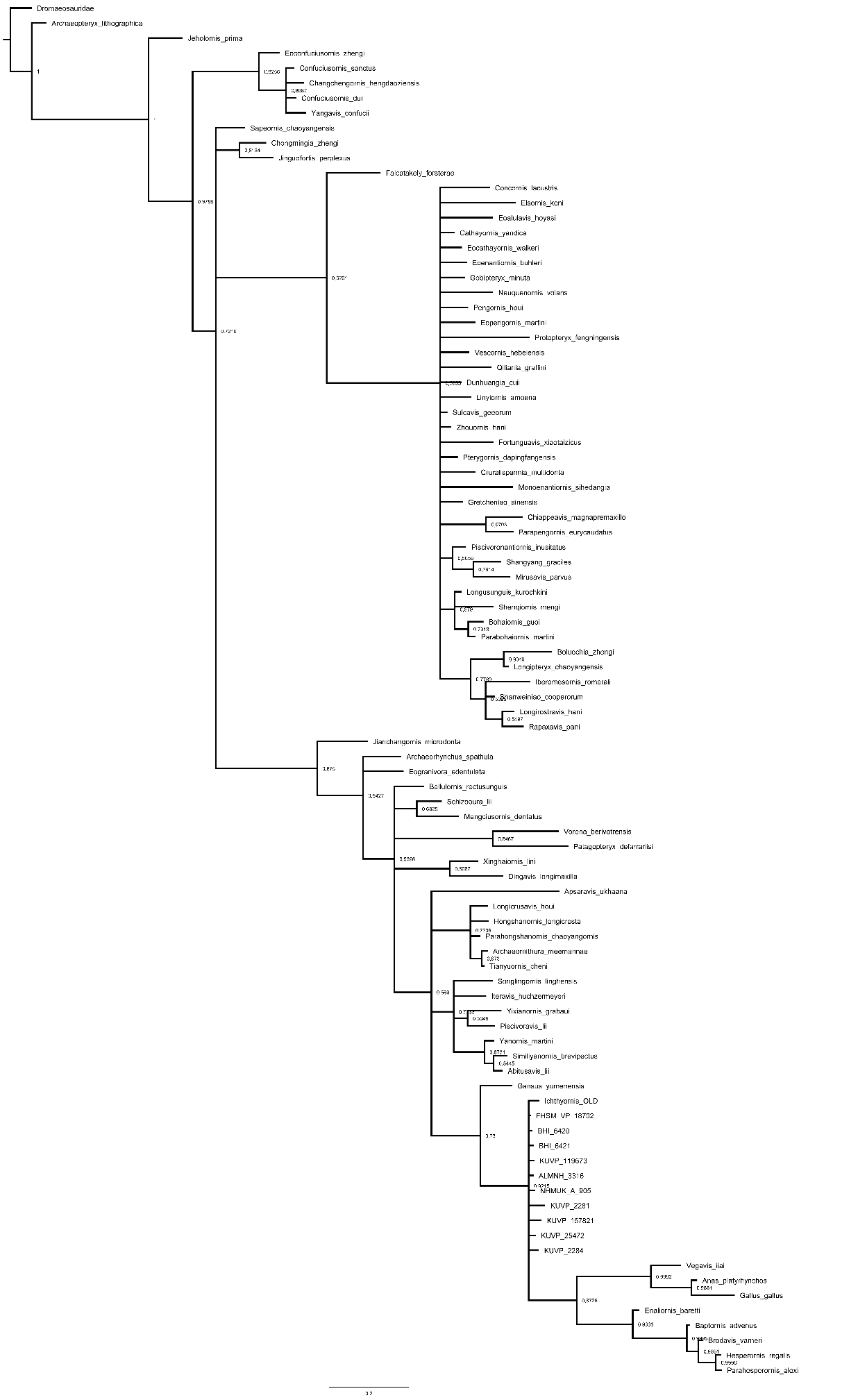
**

**Supplemental Tree 8**

**
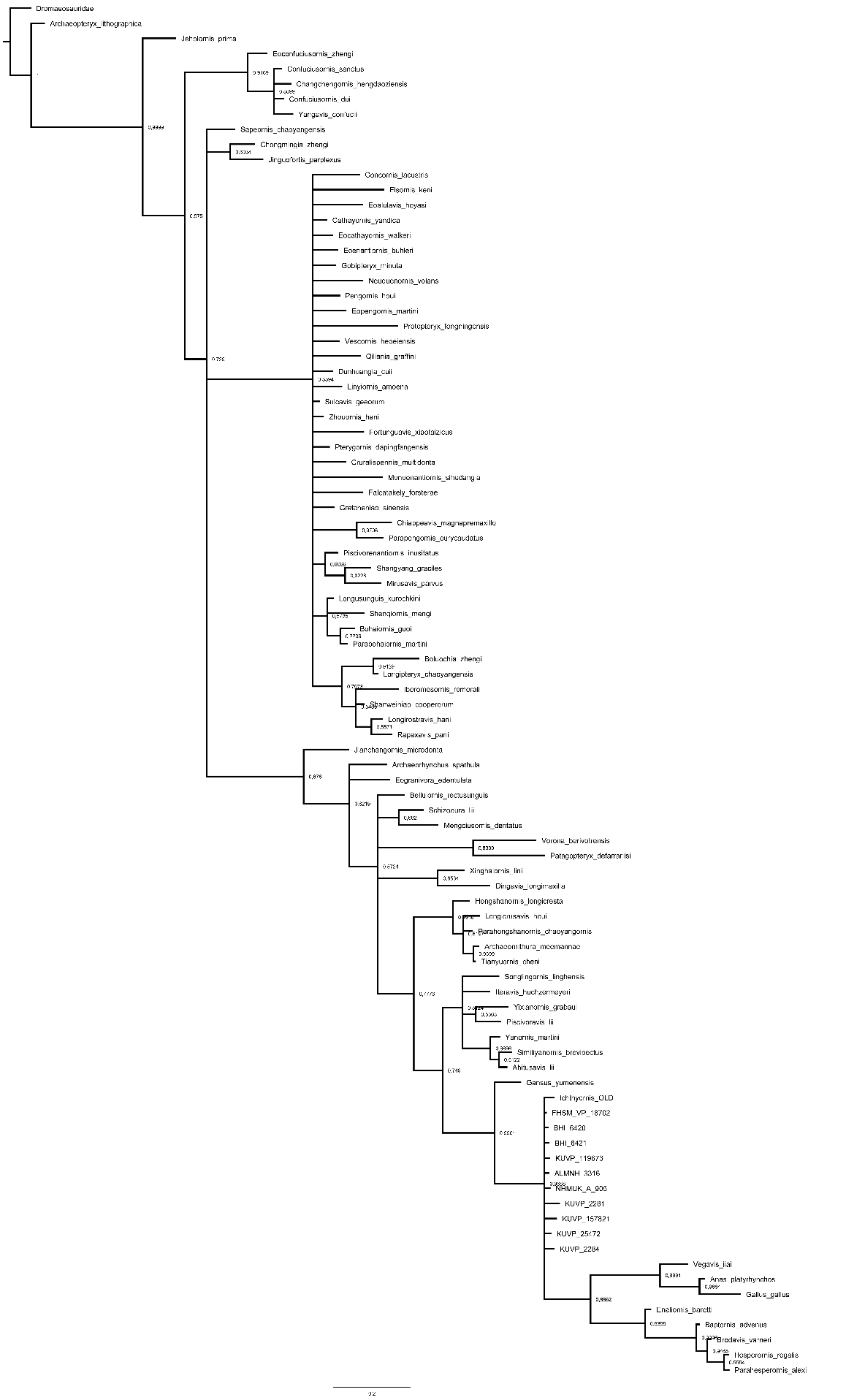
**

**
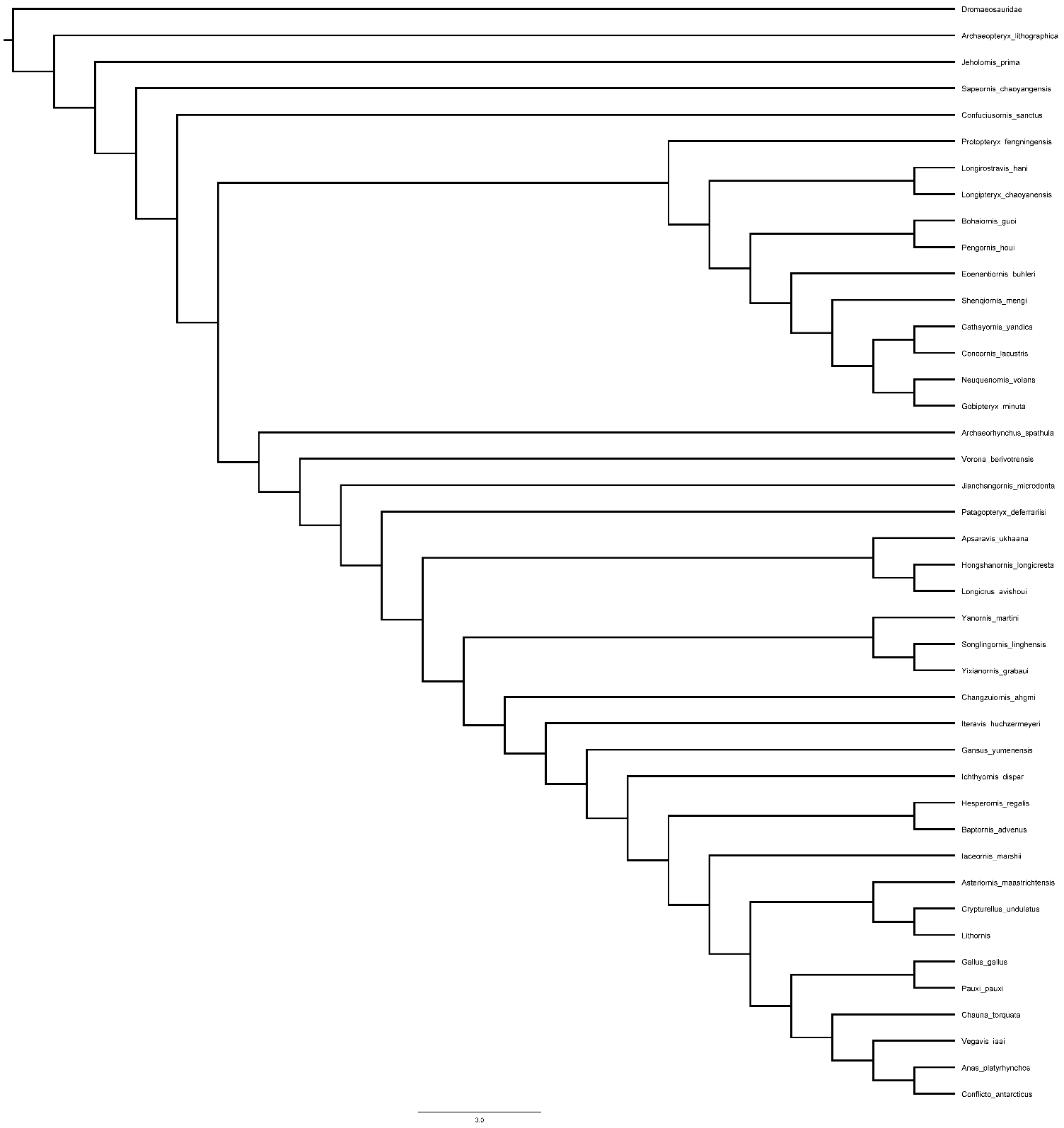
Supplemental Tree 9**

**Supplemental Tree 10**

**
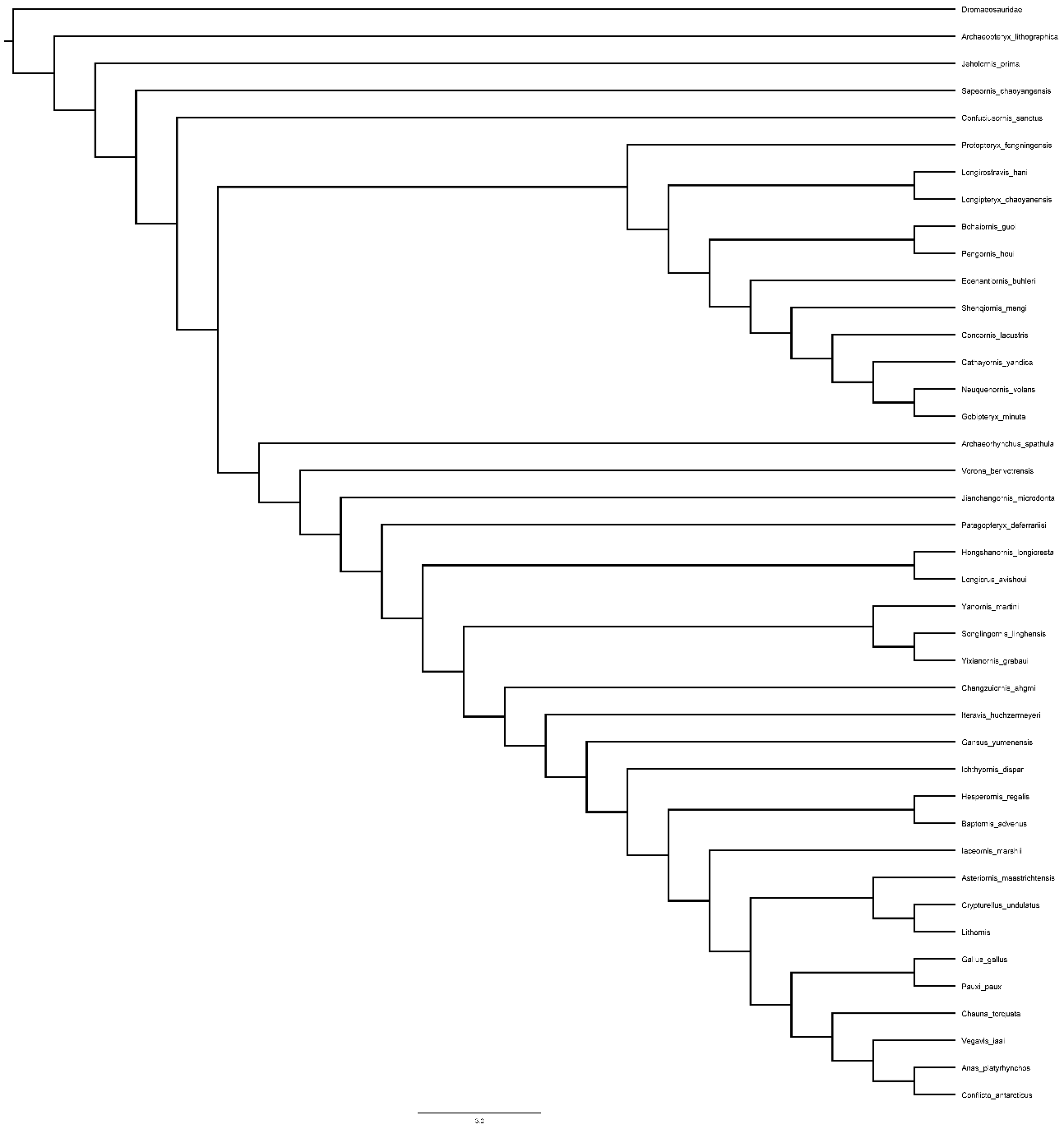
**

**
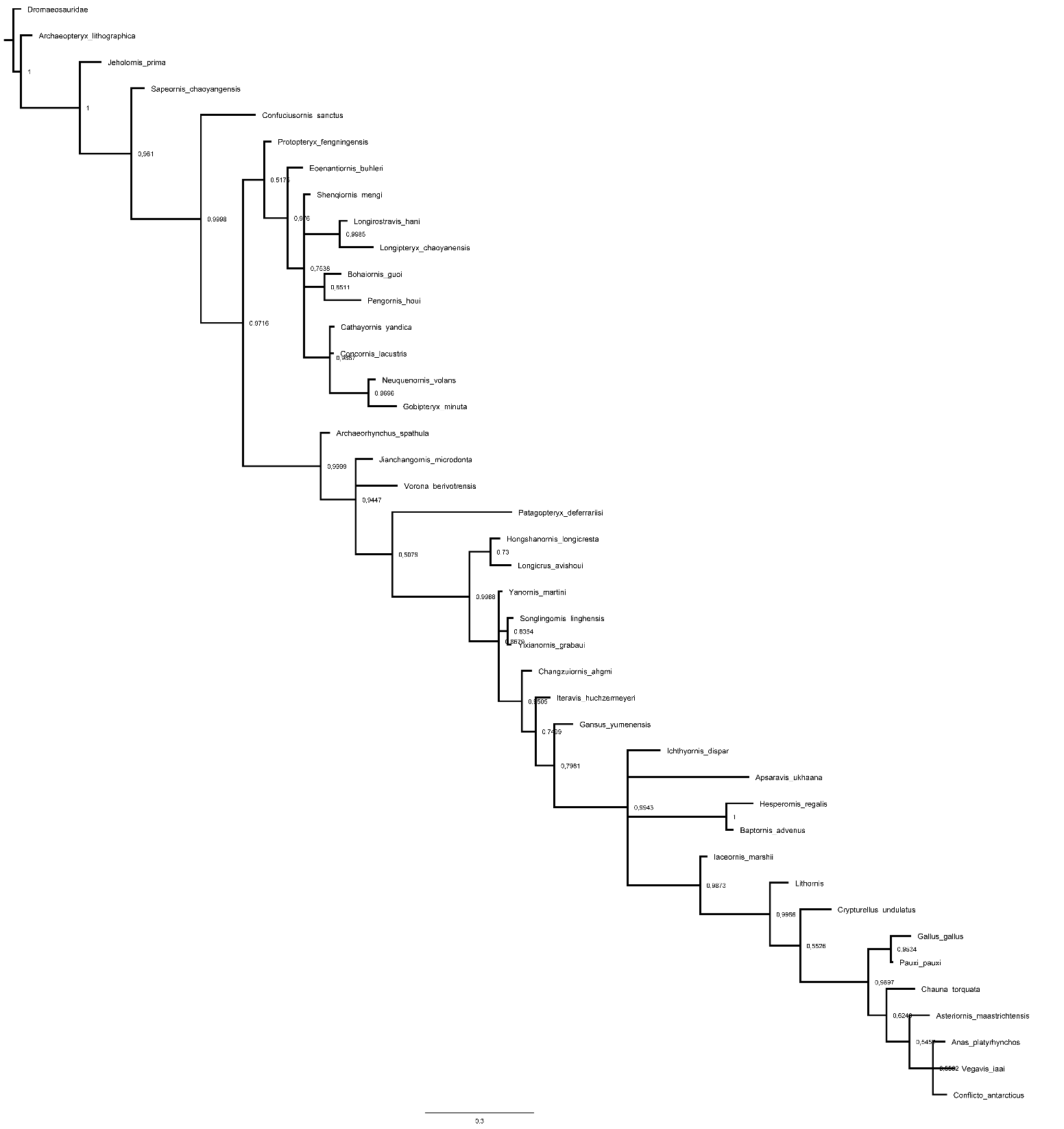
Supplemental Tree 11**

**
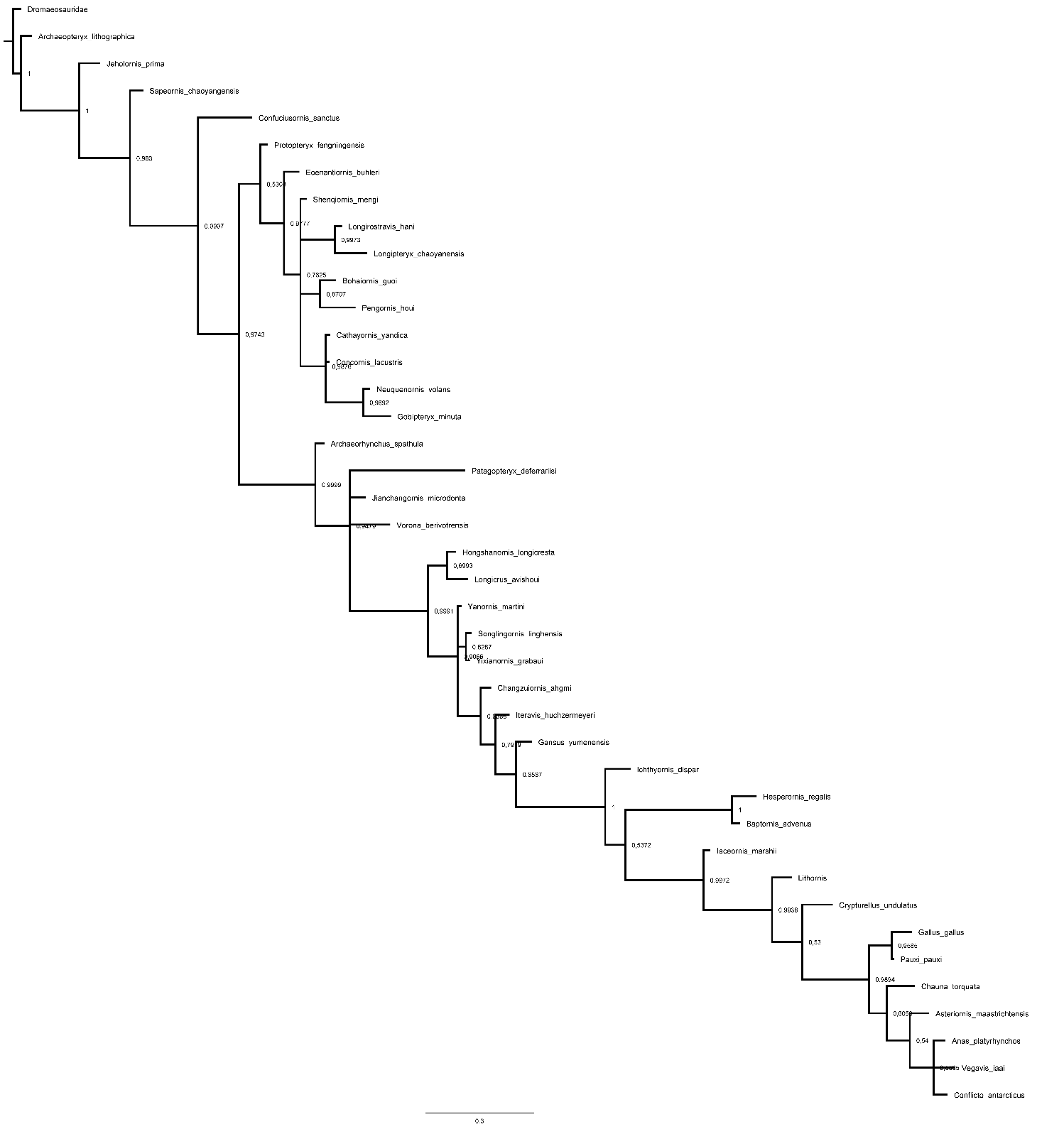
Supplemental Tree 12**

**Supplemental Tree 13**

**
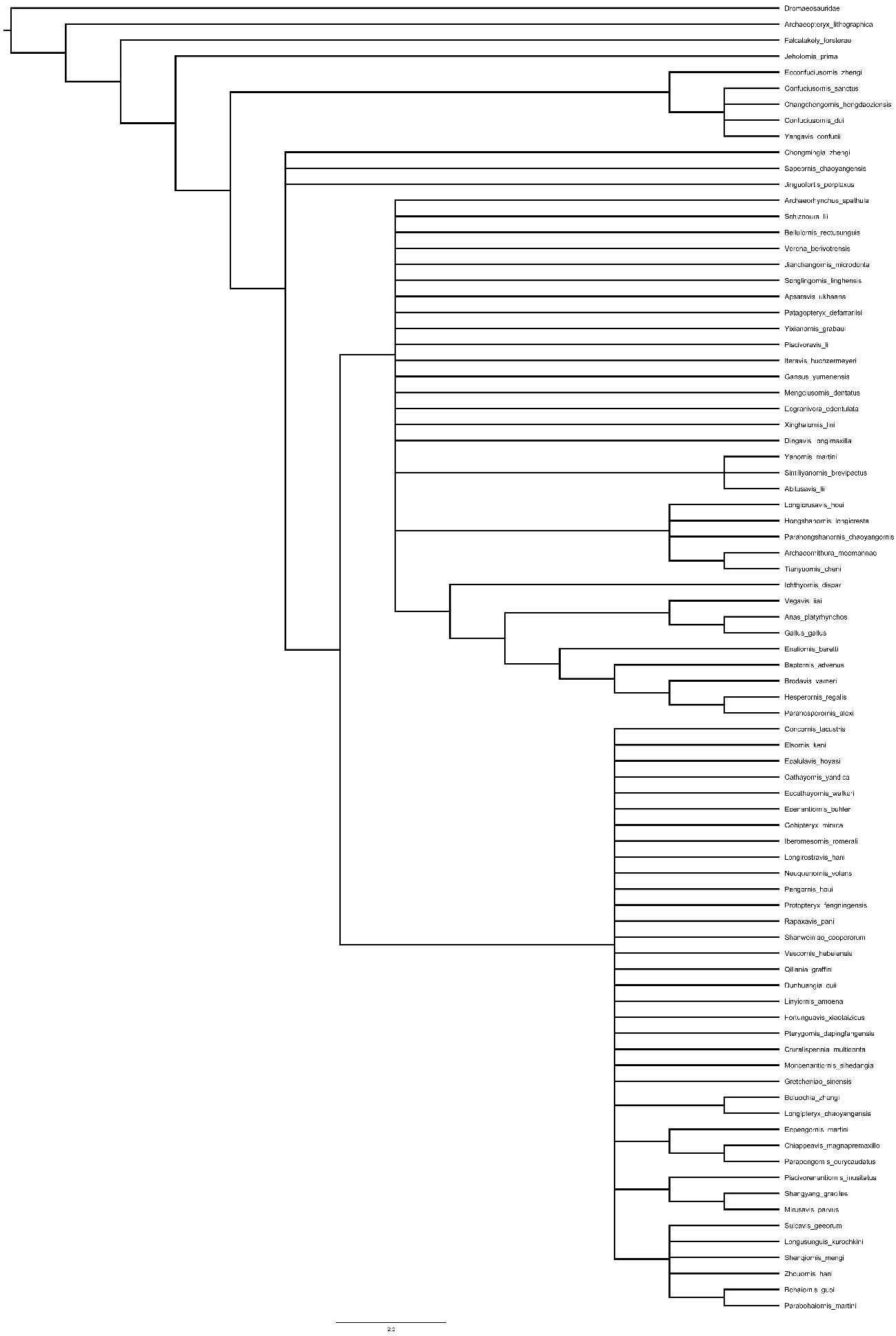
**

**
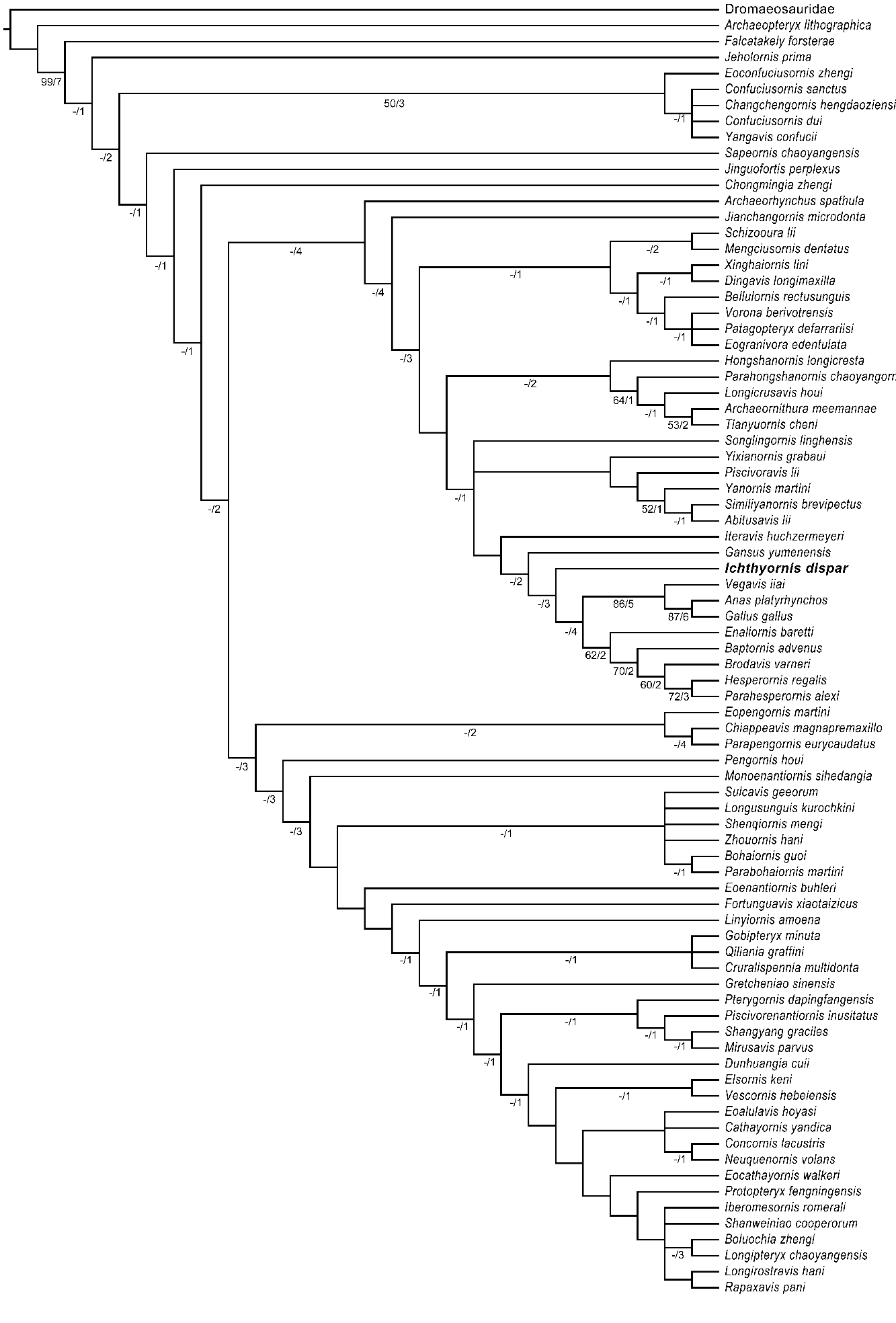
Supplemental Tree 14**


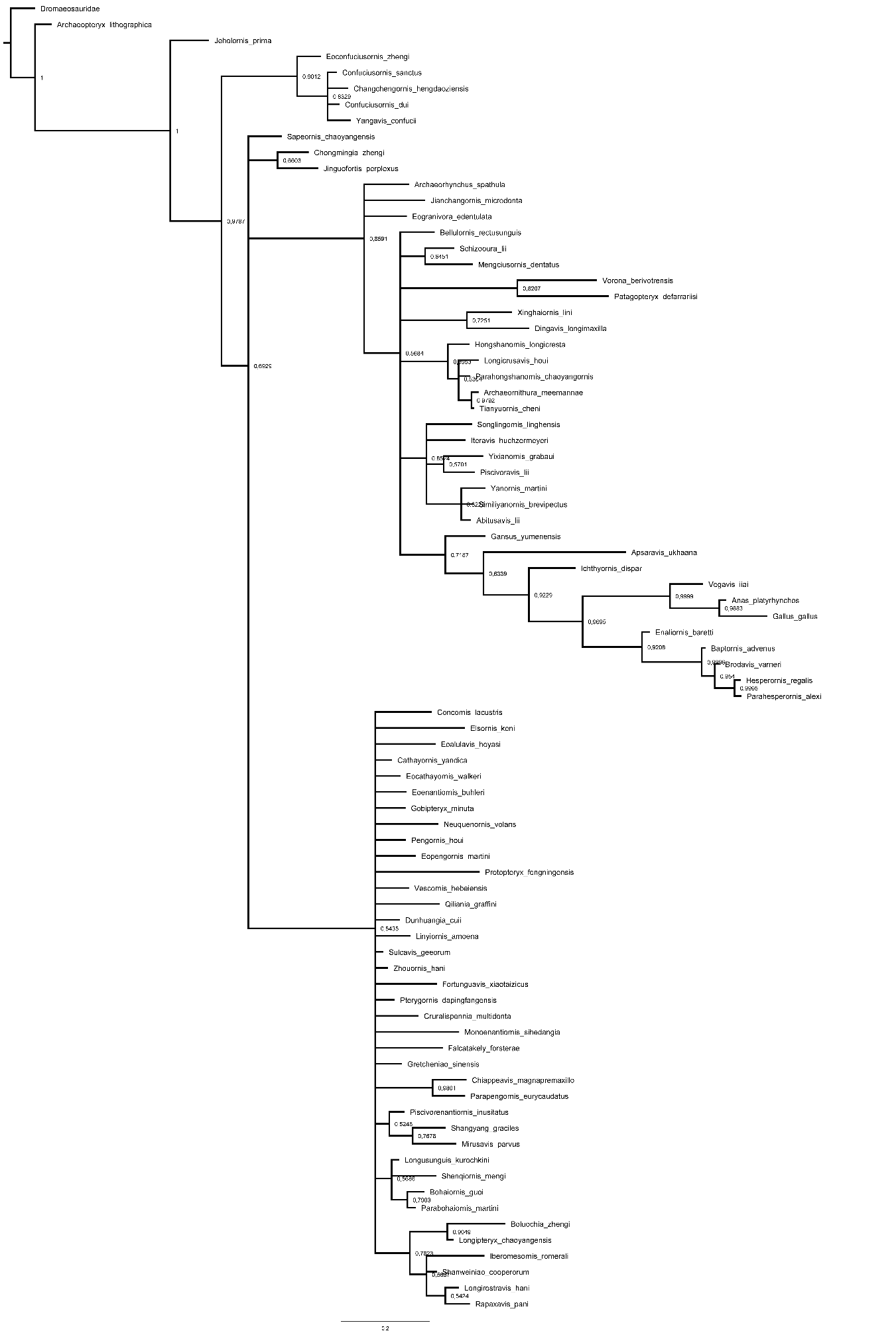
**Supplemental Tree 15**

**
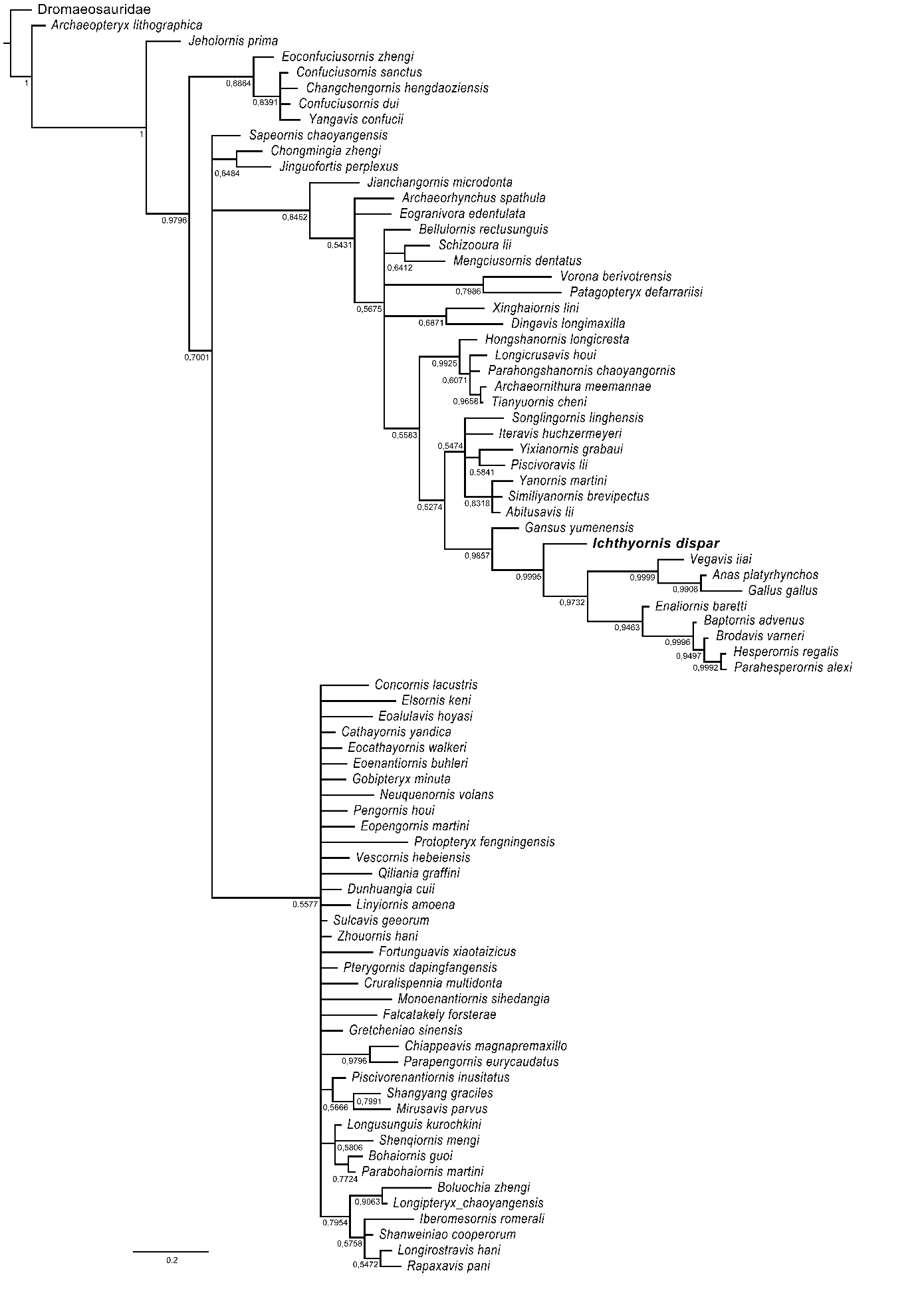
Supplemental Tree 16**

| **Species** | **Specimen** | **Hum. Length** | **Ulna Length** | **Radius Length** | **CMC length** | **Phalanx II:1** | **Phalanx II:2** | **Manus lentgh** | **Femur length** | **Tibiotarsus length** | **TMT length** | **Max pedal phalanx length** | **Max pedal digit length** | **Brachial Index** |
| --- | --- | --- | --- | --- | --- | --- | --- | --- | --- | --- | --- | --- | --- | --- |
| *Anser albifrons* | UMZC 242.EA | 150.47 | 144.51 | 140.21 | 86.47 | 37.49 | 36.87 | - | 71.8 | 130.58 | 72.96 | 28.36 | 77.25 | 1.04 |
| *Anas platyrhynchos* | UMZC 224 | 97.83 | 83.2 | 76.2 | 57.7 | 22.48 | 19.1 | 98.2 | 55.72 | 96.72 | 50.85 | 24.03 | 55.36* | 1.18 |
| *Gallus gallus* | UMZC 402 | 70.5 | 69.74 | 63.95 | 34.94 | 14.34 | 14.18 | 62.31 | 82.29 | 115.12 | 75.49 | 17.12 | 42.84* | 1.01 |
| *Fulica atra* | UMZC 298.a | 82.24 | 72.37 | 67.73 | 43.07 | 16.13 | 15.64 | 74.48 | 60.09 | 110.28 | 63.51 | 29.12 | 89.1* | 1.14 |
| *Sterna sumatrana* | UMZC 269.BA | 44.62 | 51.7 | 48.54 | 28.6 | 16.61 | 15.79 | 55.2 | 21.61 | 37.54 | 16.88 | - | 20.31 | 0.86 |
| *Chroicocephalus novaehollandiae* | UMZC 274.c | 84.95 | 94.79 | 91.12 | 49.92 | 23.7 | 20.77 | 88.12 | 40.61 | 83.03 | 53.37 | 17.37 | 42.85 | 0.90 |
| *Puffinus lherminieri* | UMZC 284.AA | 64.51 | 65.17 | 60.37 | 33.83 | 17.55 | 19.11 | 67.84 | 23.4 | 60.58 | 36.65 | - | 42.21 | 0.99 |
| *Phaethon lepturus* | UMZC 261.CA | 80.27 | 87.44 | 82.41 | 42.07 | 24.27 | 21.23 | 76.09 | 30.34 | 43.82 | 27.59 | - | 37.08 | 0.92 |
| *Podiceps auritus* | UMCZ 206.B | 76.11 | 67.95 | 67.34 | 33.45 | 14.41 | 10.07 | - | 33.16 | 85.56 | 43.39 | 18.56 | - | 1.12 |
| *Podica senegalensis* | UMCZ 209.A | 56.64 | 43.17 | 38.96 | 37.58 | 13.25 | 10.97 | 59.81 | 49.57 | 80.09 | 45.12 | 20.47 | - | 1.31 |
| *Gavia arctica* | UMCZ 203 | - | 150.37 | 148.53 | 96.04 | 29.88 | 26.22 | - | 57.07 | - | 88.08 | 54.46 | - | - |
| *Fratercula artica* | UMCZ 191.B | 61.29 | 48.94 | 46.96 | 32.12 | 14.41 | 17.54 | - | 36.21 | 62.11 | 26.38 | 12.08 | - | 1.25 |
| *Oceanodroma leucorhoa* | UMCZ 287.D | 34.93 | 34.34 | 33.92 | 19.78 | 9.75 | 10.45 | - | 15.58 | 36.59 | 22.9 | 8.44 | 22.84 | 1.02 |
| *Rhyncops flavirostris* | UMCZ 268.a | 63.67 | 76.9 | 74.98 | 37.25 | 22.03 | 21.36 | - | 28.38 | 45.91 | 24.79 | 6.83 | - | 0.83 |
| *Larus fuscus* | UMCZ 274.1 | 118.66 | 132.34 | 128.36 | 68.14 | 32.28 | 28.66 | - | 51.45 | 101.67 | 57.51 | 18.98 | 48.44* | 0.90 |
| *Chlidonias niger* | UMCZ 271.B | 41.97 | 50.49 | 48.91 | 27.83 | 16.28 | 16.04 | - | 19.56 | 35.01 | 17.47 | 6.43 | 10.93 | 0.83 |
| *Alca torda* | UMCZ 187.AA | 58.8 | 46.3 | 44.11 | 32.25 | 16.32 | 15.74 | - | 35.04 | 57.28 | 25.51 | 12.96 | - | 1.27 |
| *Uria sp.* | UMCZ 192.A | 91.13 | 76.98 | 73.13 | 49.97 | 24.68 | 27.2 | - | 51.77 | 94.97 | 40.32 | 18.71 | 62.06 | 1.18 |
| *Charadrius rubricollis* | UMCZ 320.I | 39.69 | 41.36 | 34.47 | 23.84 | 11.85 | 9.02 | - | 27.18 | 47.1 | 27.51 | 7.64 | 20.84 | 0.96 |
| *Rallus striatus* | UMCZ 304.A | 49.16 | 44 | 41.18 | 28.44 | 9.13 | 10.29 | - | 49.88 | 69.18 | 41.68 | 14.17 | 39.51* | 1.12 |
| *Sterna hirundo* | Uncatalogued | 75.81 | 84.99 | 82.31 | 46.35 | 21.81 | 20.24 | - | 35.41 | 70.46 | 46.34 | 14.8 | - | 0.89 |

**Supplemental table 1. Forelimb and hindlimb measurements of comparative extant bird specimens.** Asterisks (*) denote measurements that might be unreliable due to breakage or surrounding soft tissue but are included for completeness purposes. All measurements are in mm. - = not measurable.
